# *In situ* microwave-assisted solvothermal synthesis *via* morphological transformation of ZnCo_2_O_4_ 3D nanoflowers and nanopetals to 1D nanowires for hybrid supercapacitors†

**DOI:** 10.1039/d0ra09507a

**Published:** 2021-02-03

**Authors:** Ganesh Koyyada, Nadavala Siva Kumar, Ibrahim H. Al. Ghurabi, Mourad Boumaza, Jae Hong Kim, Koduru Mallikarjuna

**Affiliations:** School of Chemical Engineering, Yeungnam University 280 Daehak-ro Gyeongsan-si Gyeongsangbuk-do South Korea; Department of Chemical Engineering, King Saud University P. O. Box 800 Riyadh 11421 Saudi Arabia; Department for Management of Science and Technology Development, Ton DucThang University Ho Chi Minh City Vietnam koduru.mallikarjuna@tdtu.edu.vn; Faculty of Applied Sciences, Ton DucThang University Ho Chi Minh City Vietnam

## Abstract

Over recent decades, the conversion of energy and its storage have been in the lime light due to the depletion of fossil resources. The electrochemical energy storage devices like supercapacitors and batteries, and their materials and fabrication methods have been extensively evaluated, which is the best solution for the energy crisis. Herein, zinc cobaltite (ZnCo_2_O_4_; ZCO) nanostructures grown on nickel (Ni) foam by microwave-assisted solvothermal fabrication for hybrid supercapacitors are reported. Two different structures/samples, ZCO-15/Ni (nanoflowers) and ZCO-30/Ni (nanowires), were obtained by simply adjusting the reaction time. The electrochemical and physicochemical properties of the as-prepared samples were systematically determined. Particularly, ZCO-15/Ni exhibits excellent structural stability due to its dual morphologies: nanoflowers and nanopetals, and exhibits a large electroactive surface area (25.61 m^2^ g^−1^), pore diameter (48.38 nm), and robust adhesion to Ni foam, enabling ion and electron transport. ZCO-15/Ni foam electrode delivers an excellent specific capacity of 650.27 C g^−1^ at 0.5 A g^−1^ and admirable cyclic performance of 91% capacitance retention after 5000 cycles compared to ZCO-30/Ni electrode. The excellent electrochemical performance of ZCO makes them promising electrode materials for batteries, hybrid supercapacitors, and other alternative energy storage applications.

## Abstract 2

Microwave-assisted solvothermal fabrication of ZnCo_2_O_4_ nanostructures for energy storage applications like supercapacitors and batteries.

## Graphical abstract

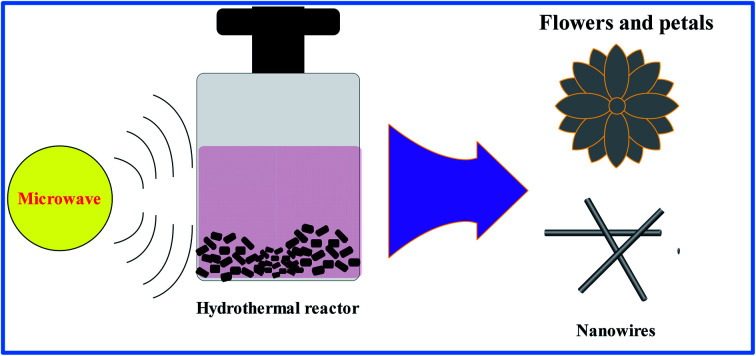

## 1. Introduction

Environmental change and consumption of petroleum products have unequivocally impacted the normal biological system and human economies (bio-economics). In order to overcome these difficulties to create and design low-cost renewable sources and ecologically friendly storage and conservation of energy-related devices to encounter the increasing market demand for the improvement of convenient electronic and electric devices and vehicles.^1–3^ Supercapacitors (SCs) and li-ion batteries (energy storage) and fuel cells (conversion) are the most promising alternative technologies for energy and environmental applications.^4–6^ Particularly, SCs are also called electrochemical capacitors or ultracapacitors, and are capable of providing a higher power density with an improved long-life than batteries and storing more than the conventional energy storage devices.^7–9^ Generally energy storage of the SCs depend on ionic adsorption (for electrical double-layer capacitors, EDLCs) or fast surface redox reactions (for pseudocapacitors).^10–12^ The combined electrode of EDLCs and pseudocapacitors form asymmetric supercapacitors, and they can extend the cell voltage.^13^ Besides, the combination of supercapacitors (EDLCs) and battery-type electrodes led to one more hybrid called supercapattery (supercapacitor + battery) or hybrid supercapacitor, which performs as the power source and energy source.^14^ In general, transition metal oxides (TMOs) display high electrochemical performance characteristics by a faradaic redox reaction.^15^ The zinc-, nickel-, cobalt-, iron-based oxides of energy storage-type materials that exhibiting Faradaic redox reaction. Cobalt-based oxides, with their advantages of great theoretical specific capacity and significant redox response, are an excellent battery-type cathode material for hybrid supercapacitor applications.^16,17^ Also, spinel metal cobaltites such as NiCo_2_O_4_,^18^ MnCo_2_O_4_,^19^ CuCo_2_O_4_,^20^ MgCo_2_O_4_,^21^ and ZnCo_2_O_4_ ^22^ are favorable electrode materials for hybrid supercapacitors because of their low electrical resistivity and excellent redox performance than the pristine metal oxides. The zinc cobaltite (ZnCo_2_O_4_; ZCO) has been viewed as a capable electrode material for supercapacitors,^23^ Li-ion batteries,^24^ and electrocatalysts^25^ because of cost-benefit and scalable alternative; moreover, it has several benefits such as low price, abundant resources and environmental friendliness. The report says that spinel ZCO has improved electrical properties and the electrochemical activity is greater than that of ZnO or Co_3_O_4_.^26^ Therefore, it is anticipated to furnish a better redox response, including more contribution from Zn and Co ions, than those of single metal zinc oxide and cobalt oxide.^27^ These striking characteristics are a big advantage for using supercapacitors with high efficiency. In order to improve the electrochemical performance of hybrid supercapacitors, one has to design electrodes with abundant electroactive sites and high transfer rates and for ions of the electrolyte and electrons participating in the faradaic reactions simultaneously.^1,28^ Recently, various morphologies of ZCO nano/microstructures on the surface of various conductive substrates like carbon cloth, FTO, ITO, and Ni foam were prepared^29,30^ and used directly as integrated electrodes for hybrid supercapacitors. The advantages of preparing electrodes through this novel method are as follows: the use of polymer binders and conductive additives can be avoided; the tedious procedure of ordinary electrode preparation can be simplified; and ordered nanoarrays on a conductive substrate can provide a more effective surface area for materials.^28,31^

In this work, we strategically prepared mesoporous ZCO nanostructures on the surface of a conducting substrate (Ni foam) through the microwave-assisted solvothermal synthesis method. By altering the microwave reaction time, we obtained two different ZCO morphologies: nanoflowers and nanowires. The two electrodes were subjected to CV, GCD, and EIS analyses to assess the electrochemical performance. The ZCO nanoflowers on Ni foam exhibited a large integral area indicated by the CV curve, higher time response from the GCD curves, and lower internal and charge-transfer resistance, which leads to high electrochemical performance compared to others. The outcomes suggest that ZCO nanostructures grown directly on Ni foam are promising electrode candidates for hybrid supercapacitors.

## 2. Experimental procedure

### 2.1 Fabrication of ZCO nanoflowers and nanowires on Ni foam

The procured chemical reagents were of analytical grade and used without further purification. Before deposition, the surface oxide layer on the Ni foam (1.5 × 4 cm^2^) was cleaned with 1 M HCl solution, absolute ethanol, and DI water by ultrasonication. The synthesis of ZCO supported on Ni foam was conducted using a microwave-assisted synthesis method using a hydrothermal reactor. Calculated amount of Zn(NO_3_)_2_·4H_2_O (2 mM), Co(NO_3_)_2_·4H_2_O (4 mM), NH_4_F (10 mM), and urea (20 mM) were dissolved in 20 mL of DI water and 20 mL of absolute ethanol (1 : 1) and stirred for 30 min to get a pink colored solution. Then, the treated Ni foam was submerged in the pink-colored precursor solution, and the solution was placed in an autoclave, properly sealed, and placed in a microwave synthesis chamber for 15 min at a maximum temperature of 180 °C, which was monitored by an infrared temperature sensor. After the reaction, it was cooled to room temperature and the Ni foam was taken out, washed several times with DI water and ethanol, and then dried at 70 °C for 12 h. Lastly, the dried sample was annealed at 400 °C for 4 h (@5 °C min^−1^) to obtain ZnCo_2_O_4_ nanostructures on Ni foam (discussed in the FE-SEM section) and labeled as ZCO-15/Ni. For comparison, the authors also prepared another sample with similar synthesis conditions except for the microwave reaction time of 30 min and labeled it as ZCO-30/Ni.

## 3. Results and discussion

### 3.1 XRD

**Fig. 1:**
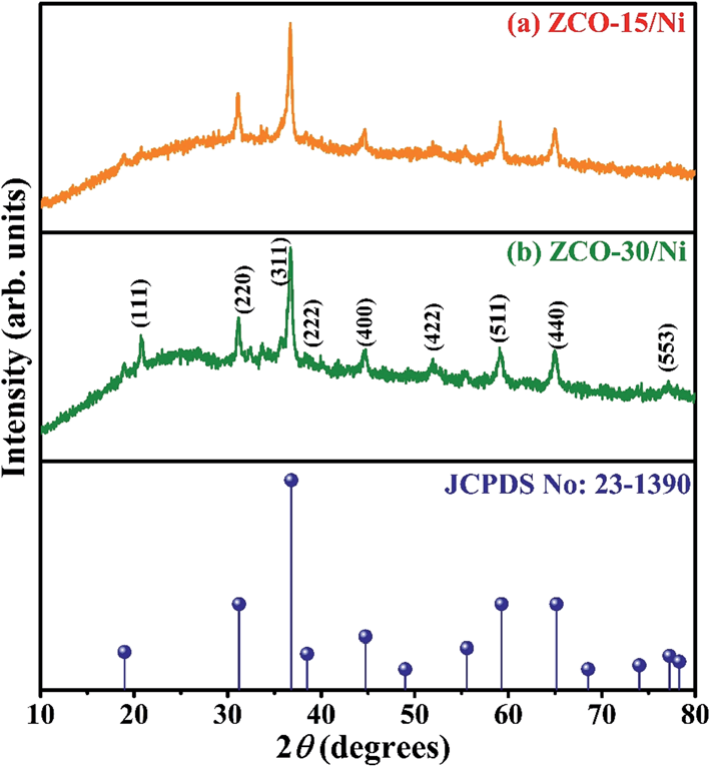
XRD patterns of ZCO-15/Ni (a) and ZCO-30/Ni (b) samples scratched out from the Ni foam and compared with standard JCPDS data.

The XRD patterns of ZCO-15/Ni and ZCO-30/Ni nanostructures (see the FE-SEM section) scratched from the Ni foam are depicted in Fig. 1. For both the samples, the diffraction peaks are centered at 2*θ* values around 18.80, 31.19, 36.78, 38.80, 44.58, 55.58, 59.27, and 65.09°, which match with the planes of (111), (220), (311), (222), (400), (422), (511), and (440) standard ZCO crystal (JCPDS no: 23-1390),^27,31^ respectively. No impurities were observed in both the samples, indicating that pure ZCO samples were produced. The crystallite size (*D*) of the samples were evaluated from the (311) plane diffraction peak with Scherrer equation: *D* = 0.9*λ*/(*β* cos *θ*), where *λ* is the monochromatic wavelength of the X-ray (1.54056 Å), *θ* is the Bragg diffraction angle, and *β* is the half-peak width of the corresponding diffracted peak. The average crystallite sizes of ZCO-15/Ni and ZCO-30/Ni were about 18.8 and 16.9 nm, respectively.

### 3.2 FE-SEM

**Fig. 2:**
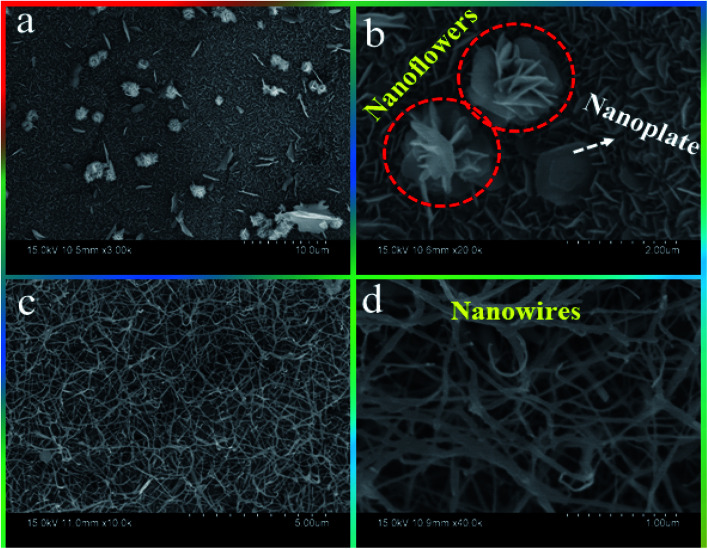
FE-SEM morphologies of (a and b) ZCO-15/Ni and (c and d) ZCO-30/Ni.

The morphology, like the shape and size, of the prepared electrode material would significantly affect the electrochemical properties. In this case, the authors prepared two different morphologies by changing the microwave reaction time (see the Experimental section) and are shown in Fig. 2. Remarkably, ZCO-15/Ni displays dual-morphology as shown in Fig. 2(a). Firstly, interconnected ZCO one-dimensional (1D) nanoplates were homogeneously grown on the surface of the Ni foam (Fig. 2a and b). The obtained morphology will bear the cost of a huge contact region for the redox response.^32^ Secondly, hierarchical 3D ZCO nanoflowers were randomly developed on the nanoplates supported by Ni foam. These ZCO nanoflowers contain several petal-like nanoplates that are basic building blocks for the formation of nanoflowers *via* the self-assembly process shown at higher magnification FE-SEM (Fig. 2(b)). Besides, ZCO nanoflowers can be used as bridges to reinforce the underlying ZCO nanoplates and reduce the effects of material volume expansion and fracture.^33^ Overall, this dual morphology of ZCO-15/Ni offers a higher BET surface area (25.61 m^2^ g^−1^; discussed in the BET section) and also prevents structural damage during the charging/discharging process. Fig. 2(c and d) shows the FE-SEM images for ZCO-30/Ni sample and exhibits 1D nanowires with different diameters that are uniformly distributed on the skeleton of Ni foam.

### 3.3 TEM

**Fig. 3:**
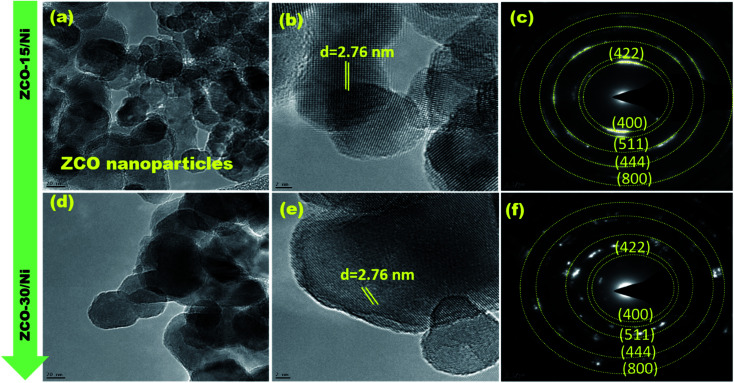
TEM (a and d), HR-TEM (b and e), and SAED patterns (c and f) for ZCO-15/Ni and ZCO-30/Ni, respectively.

The morphology and structural parameters of ZCO-15/Ni and ZCO-30/Ni samples were analyzed using HRTEM and SAED patterns. Fig. 3(a and d) and (b and e) represent low- and high-magnification images of ZCO-15/Ni and ZCO-30/Ni after ultrasonic treatment in ethanol. The prepared samples showing a number of nanoparticles are the basic building blocks that are self-assembled to form aggregates.^23^ The SAED pattern (Fig. 3(c and f)) shows a clear lattice and its crystalline properties.

From the structural and morphological studies, the possible synthesis mechanism of ZCO-15/Ni and ZCO-30/Ni was proposed. Prior to the samples grown on Ni foam, the surface of the Ni foam substrate was smooth, and after the microwave-assisted solvothermal process for 15 min, numerous nano seeds were anchored on the surface of the substrate, which is derived from the combination of Zn^2+^, Co^2+^, and OH^−^ ions. The nucleation process and formation of nanoparticles, which acts as anisotropic growth in the successive formation of petals on the Ni substrate. The flower-like structure is composed of nanopetals, and these nanopetals comprise numerous nanoparticles. Subsequently, these flower-like ZnCo-precursor nanosheets can be naturally turned into crystalline flower-like ZnCo_2_O_4_ based on the Kirkendall effect.^30^ After 30 min of microwave-assisted solvothermal reaction, nanowires were grown on the surface of the Ni foam *via* the self-assembly process.

### 3.4 XPS

**Fig. 4:**
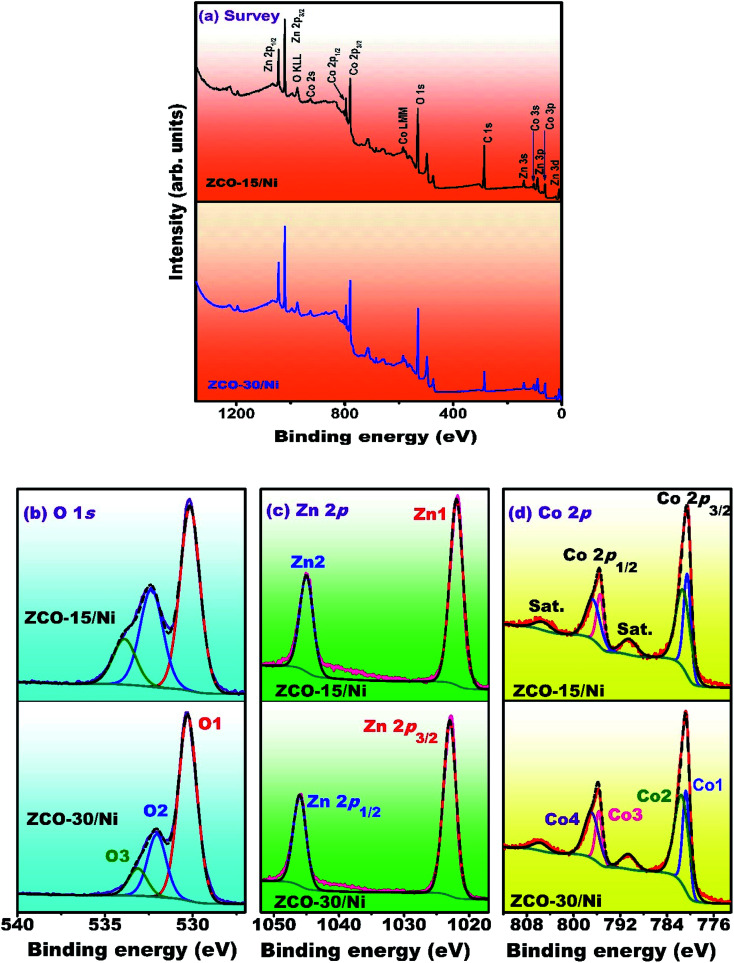
XPS spectra of ZCO-15/Ni and ZCO-30/Ni samples (a) survey scan, (b) O 1s, (c) Zn 2p, and (d) Co 2p.

The surface elemental composition and oxidation conditions of ZCO-15/Ni and ZCO-30/Ni composites were investigated *via* XPS (Fig. 4). The survey spectra for ZCO-15/Ni and ZCO-30/Ni were recorded in the 1350–0 eV range and are depicted in Fig. 4(a). For both the samples, the survey scan spectra present comparable XPS signals, indicating that they have similar composition (*i.e.* Zn, Co, and O).^34^Fig. 4(b) shows the O 1s core-level spectra of ZCO-15/Ni and ZCO-30/Ni samples exhibiting three oxygen components such as O1, O2, and O3. The binding energy positions for (ZCO-15/Ni)/(ZCO-30/Ni) were detected at 529.98/529.22 eV (O1), 531.31/531.77 eV (O2), and 532.10/533.45 eV (O3). For both the samples, the O1 peak represents metal–oxygen bonds, the O2 component corresponds to the lattice oxygen, and the O3 peak can be attributed to the surface hydroxyl groups (–OH) of the adsorbed water molecules on the surface of the materials.^35,36^ The core-level spectra (Fig. 4(c)) of Zn 2p for ZCO-15/Ni and ZCO-30/Ni exhibit two major spin–orbit peaks attributed to Zn 2p_3/2_ and Zn 2p_1/2_. The peak binding energy values of (ZCO-15/Ni)/(ZCO-30/Ni) at 1021.25/1021.36 eV (Z1) and 1044.78/1044.25 eV (Z2) were observed, indicating that Zn mainly exhibits the +2-oxidation state. Differences in the spin–orbit coupling and binding energies between the two states were assessed to be ∼23 eV, consistent with previous reports.^30,37^ Similarly the Co 2p core-level spectra for ZCO-15/Ni and ZCO-30/Ni are presented in Fig. 4(d). The irregular spectra displayed three peaks, which belonged to the +2 and +3 states of Co and a satellite peak. The satellite peaks were overlooked and +2 and +3 states were individually deconvoluted into two peaks for the comparison of the samples. For the Co^3+^ state, the spectra were fitted with two spin–orbit doublets of Co 2p_3/2_ and Co 2p_1/2_ states situated at 799.80/799.90 eV (Co1) and 794.75/794.50 eV (Co3) and similarly the peaks at 780.70/780.60 eV (Co2) and 795.90/769.10 eV (Co4) for the +2 state of Co, respectively. The binding energy difference between the two states of +2 and +3 transitions was ∼15 eV, which is well-matched with the previous research findings.^38–40^ Hence, Co had +2 and +3 states in ZCO-15/Ni and ZCO-30/Ni (Table 1).

**Table 1:** XPS results of prepared ZCO-15/Ni and ZCO-30/Ni

Sample	Peak binding energy (eV ± 0.1)/(relative atomic concentration (%))/[[FWHM] (eV)]									Elemental at%		
	O 1s			Zn 2p		Co 2p				O	Zn	Co
						Co^3+^		Co^2+^				
	O1	O2	O3	Zn1	Zn2	Co1	Co3	Co2	Co4			
ZCO-15	529.98/(40.24)/[1.20]	531.31/(10.69)/[1.20]	532.10/(7.84)/[1.21]	1021.25/(20)/[2.42]	1044.78	779.80/(4.51)/[1.21]	794.75	780.70/(14.48)/[2.72]	795.90	61.03	20	18.97
ZCO-30	529.22/(41.21)/[1.11]	531.77/(9.58)/[1.11]	533.45/(8.56)/[1.11]	1021.36/(19.84)/[2.38]	1044.25	779.90/(6.56)/[1.49]	794.50	780.60/(11.79)/[2.84]	796.10	61.92	19.06	19.02

### 3.5 BET

**Fig. 5:**
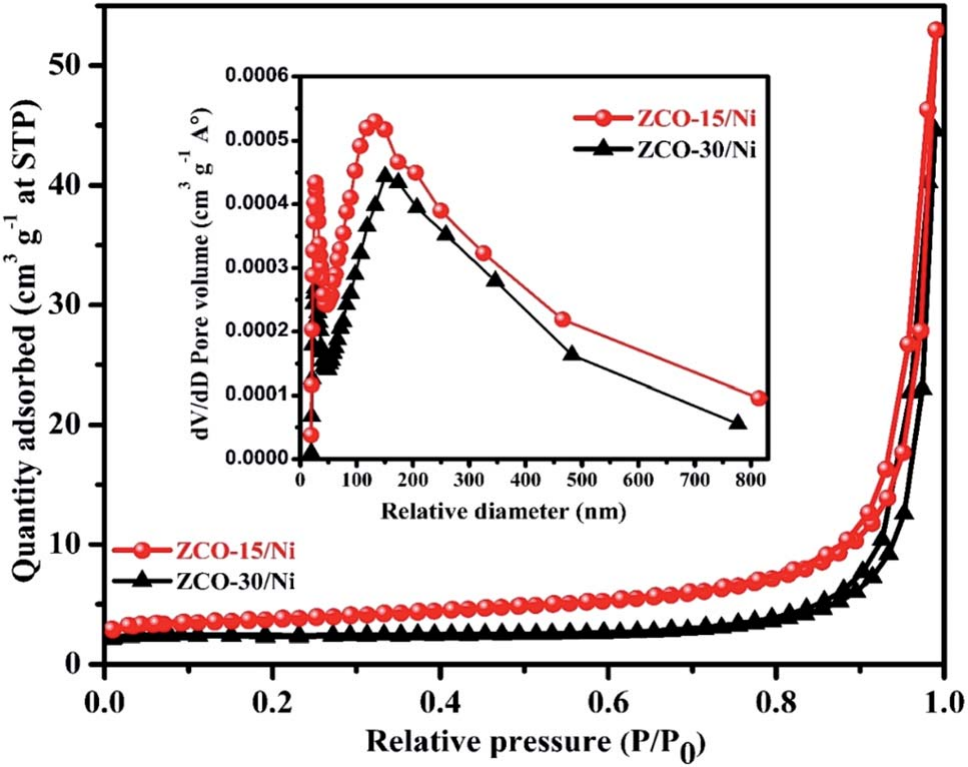
N_2_ adsorption/desorption isotherms for ZCO-15/Ni and ZCO-30/Ni and the inset figure shows pore size distribution curves for both the samples.

To investigate the surface parameters of ZCO-15/Ni and ZCO-30/Ni samples, N_2_ adsorption–desorption was studied, which are presented in Fig. 5. The two samples exhibit type-IV isotherm characteristics, which indicate the mesoporous nature of the prepared samples.^41^ ZCO-15/Ni has the largest surface area (25.61 m^2^ g^−1^) compared to ZCO-30/Ni (16.11 m^2^ g^−1^). The larger surface area of ZCO-15/Ni provides an active electrochemical site with better electrochemical reaction, raises the effective interaction area between the electrolyte and electrode, and reduces the ion/electron diffusion pathway distance in the electrolyte.^27^ Due to the small resistance, the pore volume of ZCO-15/Ni (0.079 cm^3^ g^−1^) is greater than that of ZCO-30/Ni (0.067 cm^3^ g^−1^), which effectively enhances the diffusion of the electrolyte into the active material.^27,36^ Due to this advantage, the ZCO-15/Ni material may have better electrochemical performance. Furthermore, the isotherms also provided the pore size and distribution of the respective composite materials, which were also estimated by the BJH method and the results are presented in the inset of Fig. 5. From the BJH results, the prepared composites were mesoporous. Finally, the average pore diameter was calculated to be 48.38 and 38.66 nm for ZCO-15/Ni and ZCO-30/Ni, respectively. Based on the above results (both morphological and surface characteristics), the ZCO-15/Ni sample may exhibit excellent electrochemical properties.

### 3.6 Electrochemical investigation

**Fig. 6:**
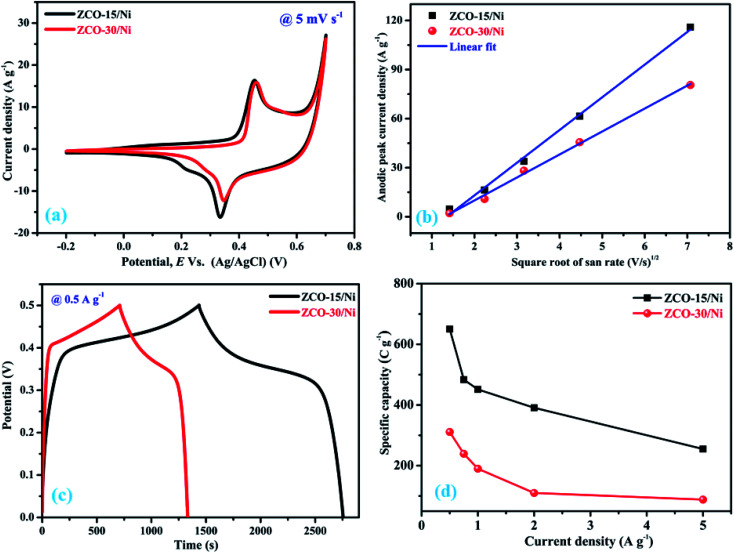
Comparison of electrochemical performance of ZCO-15/Ni and ZCO-30/Ni electrodes. (a) CV curves obtained at 5 mV s^−1^, (b) Anodic peak current density *vs.* square root of scan rate, (c) CD curves obtained at 0.5 A g^−1^, and (d) current density *vs.* specific capacity.

The electrochemical performances of the ZCO-15/Ni and ZCO-30/Ni electrodes were determined by using 3 M KOH as the electrolyte. Fig. 6(a) illustrates the assessment of characteristic CV curves of ZCO-15/Ni and ZCO-30/Ni electrodes within the voltage range of −0.2 to 0.7 V at a scan rate of 5 mV s^−1^. Particularly, the redox peak pair in the CV curves specifies that the electrochemical activity of the as-prepared electrodes originates from the pseudocapacitive behavior that can be attributed primarily to the reversible fast faradaic redox reaction. The respective redox reactions can be described by the following equation.^21,42^

1 ZnCo_2_O_4_ + OH^−^ + H_2_O ↔ ZnOOH + 2CoOOH + e^−^

The impact of Ni foam on the electrochemical performance is very limited to the whole electrode and hence was ignored. The integrated area from the CV curve of ZCO-15/Ni is significantly larger than that of ZCO-30/Ni, signifying a stronger electrochemical reaction activity of the former. This is due to the dual morphologies of the ZCO-15/Ni electrode material, higher surface area (25.61 m^2^ g^−1^), and larger pore size (48.38 nm). To additionally examine the electrochemical activity of ZCO-15/Ni and ZCO-30/Ni, we investigated the relation between peak current density (*i*) and scan rate (*v*) and followed a power law.^43,44^

2 *i* = *av*^*b*^

where ‘*a*’ and ‘*b*’ are variable parameters; when *b* = 0.5, the electrochemical process is diffusion controlled, and when *b* = 1, the electrochemical process is a non-diffusion-controlled surface redox process.^43^Fig. 6(b) shows that ZCO-15/Ni and ZCO-30/Ni have anodic peaks with *b* estimated to be 0.61 and 0.53, respectively. These values are close to 0.5, confirming that the redox process of the two electrodes is mainly diffusion-controlled.^44^

**Fig. 7:**
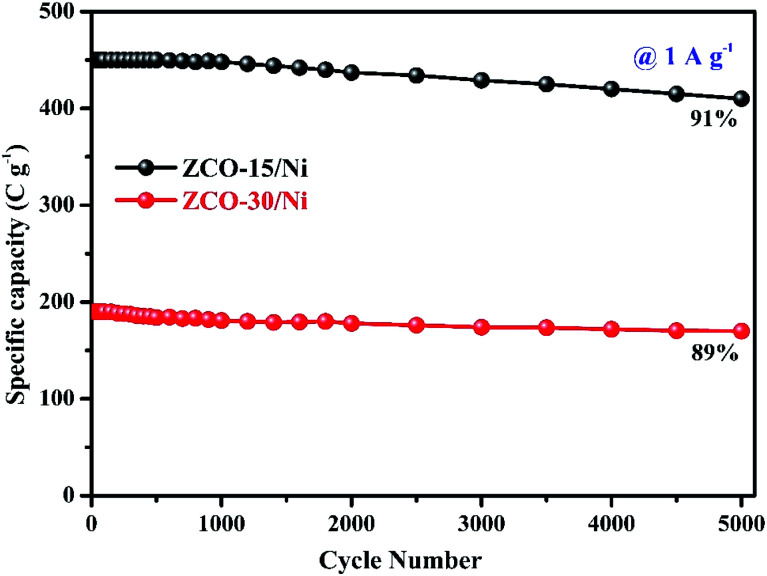
Comparison of cyclic performance for ZCO-15/Ni and ZCO-30/Ni.

Fig. 6(c) shows the evaluation of charge–discharge curves for ZC0-15/Ni and ZCO-30/Ni within a 0 to 0.5 V potential window at 0.5 A g^−1^. The potential window of CV and GCD curves are inconsistent; this is attributed to the less polarization that will occur during charging. Thus, high voltage cannot be reached as obtained in the CV curves. Practically, the potential–time curves for a current density determines high charge/discharge coulombic productivity and low polarization remarkable electrodes.^28^ ZCO-15/Ni exhibits a higher time response compared to the ZCO-30/Ni electrode. The specific capacity of the ZCO-15/Ni and ZCO-30/Ni electrodes can be calculated based on the following formula:^20,21^3
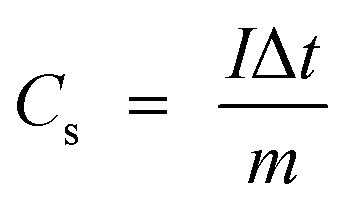
where ‘*C*_s_’ is the specific capacity in terms of C g^−1^, ‘*I*’ is the applied current, ‘Δ*t*’ is the release time, and ‘*m*’ is the mass of the dynamic material. The specific capacity instead of the specific capacitance of the ZCO-15/Ni and ZCO-30/Ni battery-type electrodes were calculated from the GCD curves to provide reasonable estimations of energy storage and discharge. The calculated specific capacity values at various current densities for both the electrodes are presented in Fig. 6(d). Remarkably, the ZCO-15/Ni electrode showed a higher specific capacity (650.27 C g^−1^ at 0.5 A g^−1^) compared to the ZCO-30/Ni electrode (311.10 C g^−1^ at 0.5 A g^−1^), suggesting that the former electrode material exhibited good charge storage performance. As the current density is enhanced, the specific capacity limit is reduced. The changes in the limit is likely brought about by the expanding voltage drop and inadequate dynamic substances engaged with redox response at high-temperature current density. The charge/discharge procedure at high current density is the utilization rate of dynamic materials is low.^1,23^Fig. 7 shows the cyclic performance of the ZCO-15/Ni and ZCO-30/Ni electrodes at 1 A g^−1^ and shows only 9% and 11% loss of the initial capacity after 5000 consecutive GCD cycles, respectively. The ZCO-15/Ni electrode has excellent cycle performance compared to ZCO-30/Ni, and demonstrates magnificent electrochemical stability and excellent application potential in battery-type supercapacitors.^45^

**Fig. 8:**
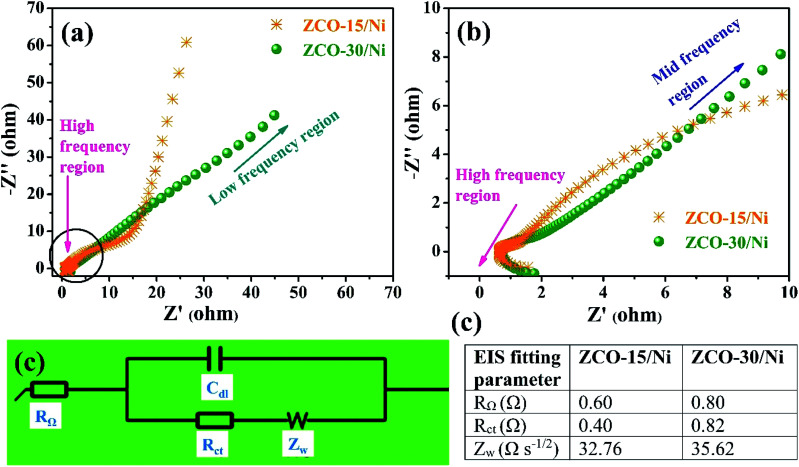
Comparison of (a) Nyquist plots of ZCO-15/Ni and ZCO-30/Ni at high to low frequency regions, (b) Nyquist plots of ZCO-15/Ni and ZCO-30/Ni at high to mid frequency regions. (c) Randles equivalent circuit model, and (d) corresponding table of EIS fitted values.

Low resistance is an important requirement for battery-type supercapacitors in practical applications, which are used as power devices. Ionic diffusion and electron transfer details of the ZCO-15/Ni and ZCO-30/Ni electrodes were investigated using EIS. Fig. 8(a) shows the Nyquist plots of ZCO-15/Ni and ZCO-30/Ni at high-to-low frequency regions, whereas Fig. 8(b) represents the magnified plot for ZCO-15/Ni and ZCO-30/Ni at high-to-mid frequency regions. For the two electrodes, the Nyquist plots at high-frequency region exposed a very small semicircle representing the charge transfer resistance (*R*_ct_) at the electrode–electrolyte interface and a straight line in the low-frequency region corresponding to the Warburg diffusion (*Z*_w_). Furthermore, the profiles were fitted in an equivalent Randles circuit model consisting of internal series resistance (*R*_Ω_), *R*_ct_, *Z*_w_, and electrical double layer capacitance (*C*_dl_) as shown in Fig. 8(c) and the obtained EIS parameters are presented in the table as shown in Fig. 8(d). From the table in Fig. 8(d), the authors concluded that the ZCO-Ni/15 electrode exhibits excellent supercapacitive performance because of the smaller *R*_Ω_, *R*_ct_, and *Z*_w_ compared to the ZCO-Ni/30 electrode.

High specific capacity, excellent cyclic performance, and high conductivity of the ZCO-15/Ni electrode confirm its suitability for practical applications. This high performance was primarily due to the following aspects: (i) Ni-foam channels and micro/nanoholes provide rich electron transport into the electrical current collector; (ii) a strategy for developing an active material on the surface of a conductive substrate (Ni foam) avoids dead volume brought about by the polymer binder and conductive carbon, significantly enhancing the use of active materials during the period of the electrochemical redox process; (iii) the dual structures of ZCO-15/Ni electrodes facilitate a huge contact surface area for the redox process, resulting in high performance.

## 4. Conclusions

ZCO nanoflowers, nanopetals (ZCO-15/Ni), and ZCO nanowires (ZCO-30/Ni) on Ni foam were successfully synthesized *via* the microwave-assisted solvothermal synthesis method. ZCO-15/Ni showed a larger surface area and pore diameter than ZCO-30/Ni, allowing for the quick transport of ions and electrons. The ZCO-15/Ni electrode achieved a maximum specific capacity of 650.27 C g^−1^ compared with ZCO-30/Ni with 311.10 C g^−1^ at 0.5 A g^−1^. After 5000 cycles, both the grown structures showed around 91% and 89% of the initial specific capacity. Low internal and charge-transfer resistance was exhibited by ZCO nanoflowers and nanopetals on Ni form. This work provides a new approach for the rational selection of the reaction time for high-performance hybrid supercapacitors.

## Conflicts of interest

The authors declare that there is no conflict of interest.

## Supplementary Material

RA-011-D0RA09507A-s001
