# Supplementary material for: In situ microwave-assisted solvothermal synthesis via morphological transformation of ZnCo2O4 3D nanoflowers and nanopetals to 1D nanowires for hybrid supercapacitors
Source: RSC Adv. 2021 Feb 3;11(11):5928–37. doi: 10.1039/d0ra09507a (PMC8694834; doi:10.1039/d0ra09507a)
Supplement: RA-011-D0RA09507A-s001 [file RA-011-D0RA09507A-s001.pdf]

Electronic Supplementary Material (ESI) for RSC Advances.
This journal is © The Royal Society of Chemistry 2021

In-situ microwave-assisted solvothermal synthesis of morphological transformation of $ZnCo_2O_4$ 3D nanoflowers and nanopetals to 1D nanowires for hybrid supercapacitors

Koyyada Ganesh [a,¶], Nadavala Siva Kumar [b,¶], Ibrahim H. Al.Ghurabi [b], Mourad Boumaza[b], Jae Hong Kim[a], Koduru Mallikarjuna [c,d,*]

[a] School of Chemical Engineering, Yeungnam University, 280 Daehak-ro, Gyeongsan-si, Gyeongsangbuk-do, South Korea.

[b] Department of Chemical Engineering, King Saud University, P.O. Box 800, Riyadh, 11421, Saudi Arabia

[c] Department for Management of Science and Technology Development, Ton Duc Thang University, Ho Chi Minh City, Vietnam.

[d] Faculty of Applied Sciences, Ton Duc Thang University, Ho Chi Minh City, Vietnam

¶The authors are equally contributed

Corresponding author Email: koduru.mallikarjuna@tdtu.edu.vn

1. Characterization

The crystallinity and facets formation of prepared ZCO/Ni were investigated through the X-ray diffractometer (XRD; PANalytical X'Pert Pro) operating at 40 kV and 30 mA with CuKα radiation (λ =1.540 Å). The morphology features like the size and shape of the grown structures were analyzed by a field emission SEM (FE-SEM). The high-resolution images were revealed through the transmission electron microscope (HR-TEM) were recorded using a field emission electron gun in Schottky mode operating at 200 kV. N2 adsorption/desorption curves were recorded using a BET surface analyzer.

Electrochemical estimations were performed utilizing a mechanized electrochemical workstation (CHI 760 E, CH Instruments, USA) utilizing a three-anode arrangement at RT. In all tests, Ag/AgCl, Pt wire, and ZCO nanoflowers/nanowires Ni foam were utilized as the reference, counter, and working terminals, separately, and 3 M KOH arrangement was used as the dynamic/active electrolyte. Cyclic voltammetry (CV) and charge/release (CD) bends were gained and electrochemical impedance spectroscopy (EIS) was performed to explore the electrochemical exhibition of ZCO/Ni tests.
